# Progesterone and selective progesterone receptor modulator regulate tumor-associated fibroblasts in uterine leiomyomas

**DOI:** 10.1016/j.gendis.2026.102041

**Published:** 2026-01-16

**Authors:** Weronika Szucio, Gabriela Milewska, Michał Szamatowicz, Weronika Lebiedzińska, Oana Lupu, Piotr Bernaczyk, Monika Leśniewska, Peilan Guo, Ilpo Huhtaniemi, Xiangdong Li, Sławomir Wołczyński, Donata Ponikwicka–Tyszko, Nafis A. Rahman

**Affiliations:** a Department of Reproduction and Gynecological Endocrinology, Medical University of Bialystok, Bialystok 15-089, Poland; b Department of Medical Pathomorphology, Medical University of Bialystok, Bialystok 15-089, Poland; c State Key Laboratory of Agrobiotechnology, China Agricultural University, Beijing 100193, China; d Institute of Reproductive and Developmental Biology, Department of Metabolism, Digestion and Reproduction, Imperial College London, London W12 0NN, UK; e Institute of Biomedicine, University of Turku, Turku 20520, Finland; f Clinical Research Centre, Medical University of Bialystok, Bialystok 15-276, Poland

Uterine leiomyomas (ULs) are benign tumors found in 60%–70% of women, with a substantial negative impact on quality of life.^1^ ULs originate from mutated smooth muscle cells, characterized by a disordered architecture, abundant extracellular matrix (ECM), and an admixture of fibroblasts.^1^ These fibroblasts are not passive components; they actively shape the tumor microenvironment (TME) by releasing cytokines. This activity can drive the formation of a highly active fibroblast subgroup known as tumor-associated fibroblasts (TAFs), which are instrumental in disease progression.^2^

The role of progesterone (P4) in uterine leiomyoma (UL) growth is well-established, involving the stimulation of cellular proliferation and ECM synthesis.^1^ Selective progesterone receptor modulators (SPRMs), such as ulipristal acetate (UA), are effective drugs, yet their mechanisms of action remain only partly understood.^1^ Unraveling the precise mechanism of SPRM's action could improve our knowledge of UL biology and lead to safer, more effective novel pharmacological therapies. This study aimed to characterize the molecular mechanisms underlying the effects of P4 and UA on TAF markers expression profile in ULs, which may reveal new insights into their potential role in ECM/TME modulation. We hypothesized that UA counteracted P4-driven tumorigenesis by altering TAF phenotype, thereby providing new insights into ECM and TME remodeling and identifying pathways for future drug development.

Growing attention is being given to the TME/ECM and the role of TAFs, as TAFs have been linked to the development and remodeling of the TME in various tumors, including ULs. We analyzed the expression profiles of selected TAF marker genes, *FAP*, *αSMA*, *S100A4*, platelet-derived growth factor receptor alpha (*PDGFRα*), and platelet-derived growth factor receptor beta (*PDGFRβ*), in UL tissues after ulipristal acetate treatment (UA-L), non-treated UL group (NT-L), and healthy myometrium (M) tissues. The expression level of *FAP* was significantly higher in NT-L compared with both normal M and UA-L (Fig. 1A). In contrast, expression levels of *αSMA* (Fig. S1A) and *S100A4* (Fig. S1B) were similar in all groups. To further investigate the effects of P4 and UA on TAF expression, we treated *in vitro* UL explants with P4, UA, and transforming growth factor beta-3 (TGFβ3) as a positive control. UA significantly down-regulated, whereas TGFβ3 up-regulated *FAP* expression level (Fig. 1B). In contrast, no significant changes were observed in the expression levels of *αSMA* (Fig. S1C) and *S100A4* (Fig. S1D) after treatment of UL explants with any of the stimulants. While P4 signaling is thought to drive UL formation through several proposed mechanisms, its exact influence on tumor biology is not yet fully understood.^1^ In this study, we demonstrated that P4 and UA differentially regulated the expression of key TAF markers in ULs. These findings align with previous reports, demonstrating that activation of the TLR4/NF-κB pathway in UL-associated fibroblasts enhances their activation state, marked by increased FAP expression and the production of collagen I and TGF-β^3^ Taken together, these findings suggest that TAFs may play an important functional role in UL pathophysiology.

**Figure 1:**
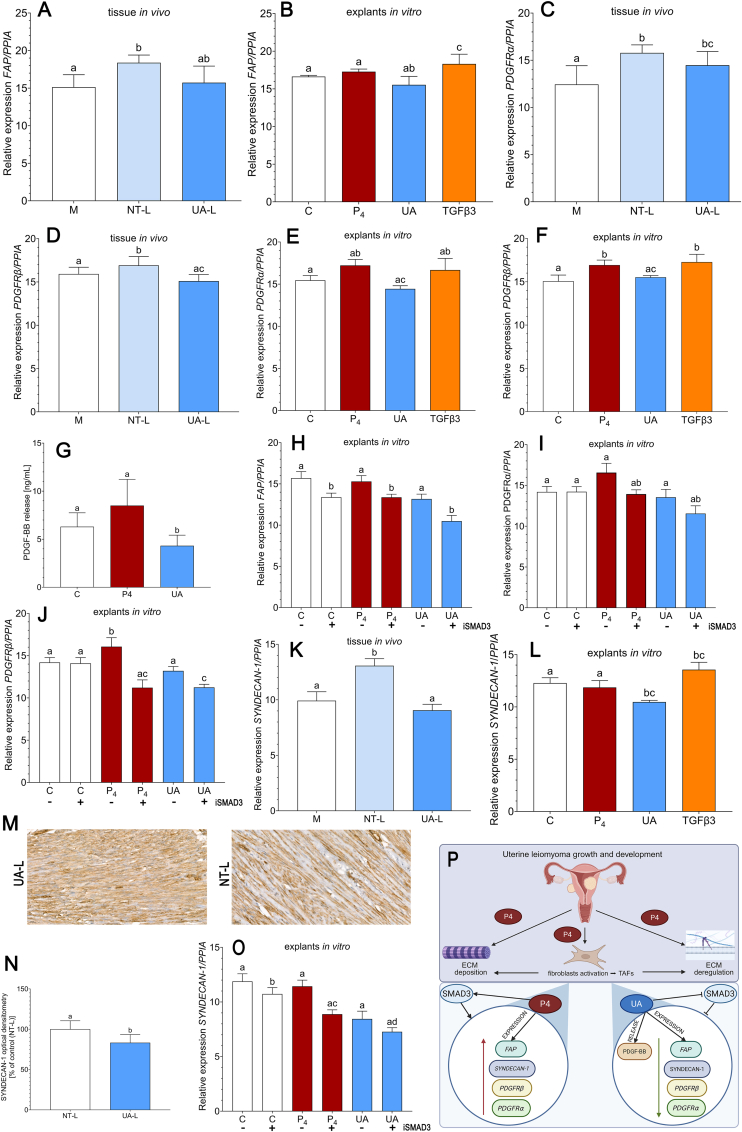
Overview of progesterone and ulipristal acetate action on tumor-associated fibroblasts markers and mediators in the uterine leiomyoma. **(A)** The quantitative polymerase chain reaction (qPCR) gene expression analyses of fibroblast activation protein (*FAP*) healthy myometrium (M) (*n* = 30), uterine leiomyoma (UL) tissue after ulipristal acetate (UA) treatment (UA-L) (*n* = 50), and non-treated group (NT-L) (*n* = 50). **(B)** UL explant culture *in vitro* treated with UA, progesterone (P4), or transforming growth factor beta 3 (TGFβ3). **(C)** The quantitative polymerase chain reaction (qPCR) gene expression analyses of platelet-derived growth factor receptor alpha (*PDGFRα*). **(D)** Platelet-derived growth factor receptor beta (*PDGFRβ*), in healthy myometrium (M) (*n* = 30), uterine leiomyomas (UL) after UA treatment (UA-L) (*n* = 50), and non-treated uterine leiomyomas (NT-L) (*n* = 50). **(E)** The qPCR gene expression profile of platelet-derived growth factor receptor alpha (*PDGFRα*). **(F)** Platelet-derived growth factor receptor beta (*PDGFRβ*), in UL explant culture *in vitro* treated with UA, progesterone (P4), or transforming growth factor beta 3 (TGFβ3). **(G)** Release of platelet-derived growth factor subunit B (PDGF-BB) levels in the control, P4, and UA-treated groups in explant culture *in vitro* is shown. **(H)** The quantitative polymerase chain reaction (qPCR) gene expression analysis of fibroblast activation protein (*FAP*). **(I)** Platelet-derived growth factor receptor alpha (*PDGFRα*). **(J)** Platelet-derived growth factor receptor beta (*PDGFRβ*), in uterine leiomyoma (UL) explant culture *in vitro* treated with UA and progesterone (P4) with or without inhibitor of SMAD3 (iSMAD3). **(K)** The quantitative polymerase chain reaction (qPCR) gene expression analyses of *SYNDECAN-1* in healthy myometrium (M) (*n* = 30), uterine leiomyoma (UL) tissue after UA treatment (UA-L) (*n* = 50), and the non-treated group (NT-L) (*n* = 50). **(L)** UL explant culture *in vitro* treated with UA, progesterone (P4), or transforming growth factor beta 3 (TGFβ3). **(M)** Immunohistochemical (IHC) staining of SYNDECAN-1 in ULs from NT-L and UA-L groups is represented. **(N)** Quantification of IHC staining of SYNDECAN-1 by Fiji (ImageJ). For each section, five randomly chosen areas were automatically analyzed with the Fiji software to assess optical density (OD). Data were expressed as percentages relative to the control untreated group (*p* < 0.05; *t*-test). Scale bar: 20 μm. **(O)** The qPCR gene expression analysis of *SYNDECAN-1* in UL explants *in vitro* after treatment with UA and P4, with or without inhibitor of SMAD3 (iSMAD3). **(P)** Tumor-associated fibroblasts (TAFs) remodel extracellular matrix (ECM) by secreting various components, cytokines, and enzymes. ECM further activates TAFs, creating a positive feedback loop. Progesterone (P4) and UA differentially regulate TAF-associated markers in uterine leiomyomas (ULs). P4 up-regulates fibroblast activation protein *(FAP),* platelet-derived growth factor receptor α/β (*PDGFRα/β*), and *SYNDECAN-1*. In contrast, UA down-regulates their expression as well as decreases platelet-derived growth factor subunit B (PDGF-BB) secretion. Additionally, these modulatory effects depend on SMAD3 signaling. Statistical differences between groups were assessed using one-way ANOVA followed by Bonferroni's post-hoc test. Bars labeled with different letters differ significantly (*p* < 0.05). C, control; NT-L, nontreated ULs; M, healthy myometrium; P4, progesterone treatment; UA-L, ulipristal acetate-treated ULs; UA, ulipristal acetate treatment; TGFβ3, transforming growth factor beta 3 treatment; iSMAD3, an inhibitor of SMAD3; ULs, uterine leiomyomas; ECM, extracellular matrix; PDGF-BB, platelet-derived growth factor subunit B; PDGFRα, platelet-derived growth factor receptor α; PDGFRβ, platelet-derived growth factor receptor β; FAP, fibroblast activation protein; TAFs, tumor-associated fibroblasts.

Abnormal remodeling and excessive ECM accumulation are hallmark features of ULs, supporting their characterization as a fibrotic disorder.^1^ Key profibrotic factors, such as TGFβ and PDGF, have been associated with driving this pathological ECM accumulation in ULs^1^ In our study, we also aimed to investigate PDGFR*s* and the platelet-derived growth factor subunit B (PDGF-BB) signaling pathway. The expression levels of *PDGFRα* (Fig. 1C) and *PDGFRβ* (Fig. 1D) were significantly higher in NT-L compared with normal myometrium and UA-L. P4 and TGFβ3 significantly up-regulated, whereas UA down-regulated the expression levels of *PDGFRα* in UL explants (Fig. 1E). *PDGFRβ* expression was also up-regulated by P4 and TGFβ3 treatments (Fig. 1F), whereas UA down-regulated its expression when compared with TGFβ3 and P4 (Fig. 1F). In addition, UA significantly decreased the secretion level of PDGF-BB in UL explants compared with P4 (Fig. 1G). These findings suggest that UA may attenuate excessive ECM accumulation in ULs by suppressing PDGF-BB-mediated fibroblast activation. Emerging evidence indicates that modulation of PDGF signaling may represent a promising therapeutic strategy for controlling UL progression and alleviating related symptoms. We hypothesized that the regulation of TAF markers (FAP, PDGFRα, PDGFRβ) by UA and P4 was mediated through SMAD3 signaling, a key transcription factor in the TGFβ pathway. Analysis of UL explants revealed that pharmacological inhibition of SMAD3 (iSMAD3) significantly down-regulated *FAP* expression (Fig. 1). Moreover, iSMAD3 completely reversed the stimulatory effect of P4 on *FAP* (Fig. 1H), *PDGFRα* (Fig. 1I), and *PDGFRβ* (Fig. 1J). Furthermore, iSMAD3 also demonstrated an additive effect to the inherent inhibitory action of UA on these markers, suggesting that combined targeting of progesterone and SMAD3 pathways may yield superior suppression of TAF activation (Fig. 1H–J).

The role of SMAD3 in regulating fibroblast activation within the TME has been demonstrated in other neoplasm models, like lung carcinoma.^4^ In non-small-cell lung carcinoma, SMAD3 has been shown to promote the formation of pro-tumoral TAFs through the macrophage–myofibroblast transition, through direct binding to genes that regulate fibroblast differentiation.^4^ Pharmacological inhibition or macrophage-specific deletion of macrophage–myofibroblast transition has been shown to strongly suppress TAF formation and delay tumor development.^4^ Taken together, the conserved role of SMAD3 underscores the significance of our findings: the opposing effects of P4 and UA on TAF marker expression in ULs are directly linked to their modulation of SMAD3 signaling, revealing a fundamental mechanistic overlap in fibroblast activation markers within UL tissue. The proteoglycan SYNDECAN-1 plays a role in cell–matrix interactions and tissue remodeling, which are important processes in tumor growth and fibroblast activation.^5^ Our data robustly demonstrated that *SYNDECAN-1* expression was significantly higher in NT-L compared with normal M and UA-L (Fig. 1K). The regulatory profile of SYNDECAN-1 aligned with a pro-fibrotic phenotype, as its expression was stimulated by TGFβ3 and, crucially, inhibited by UA in UL explants (Fig. 1L). Abundant SYNDECAN-1 staining was observed in NT-L (Fig. 1M). UA treatment markedly decreased its protein expression level (Fig. 1M and N). This positioned SYNDECAN-1 as a downstream element in the pathway targeted by UA. iSMAD3 down-regulated *SYNDECAN-1* expression in control and P4-treated UL explants (Fig. 1O). Furthermore, iSMAD3 showed an additive inhibitory effect on *SYNDECAN-1* expression with UA (Fig. 1O). The additive inhibitory effect of iSMAD3 and UA suggests that SYNDECAN-1 is a point of convergence for anti-fibrotic therapies, reinforcing its potential role as a key mediator of fibroblast activation and tissue pathology in this setting.

Recent studies have indicated that SYNDECAN-1 may regulate several ECM components, including fibronectin, collagen type 1, α-SMA, and MMPs in ULs^5^ Silencing *SYNDECAN-1* in UL cells resulted in reduced cell proliferation and decreased levels of ECM components and MMPs^5^ Furthermore, it has been shown that silencing *SYNDECAN-1* enhances the anti-proliferative effects of another SPRM, mifepristone, in human UL cells.^5^ We found here that UA significantly down-regulated *SYNDECAN-1* expression in ULs. Consequently, our findings underscore the compelling dual potential of SYNDECAN-1 as an actionable therapeutic target and a promising biomarker for treatment response in uterine leiomyomas.

ULs remain the leading indication for surgical procedures such as myomectomy or hysterectomy. This emphasizes the need for pharmacological treatment options that are both effective and fertility-preserving. Our study delineates a central mechanism underlying this need, as P4 enhanced the activation of TAF markers in ULs, whereas UA counteracts this effect (summarized in Figure 1P). We have identified SMAD3 signaling as the critical pathway mediating this opposing regulation. This novel finding suggests that the therapeutic efficacy of UA could be significantly enhanced by combining it with direct anti-fibrotic agents, representing a novel, multi-targeted strategy to suppress UL growth and mitigate the burden of surgical intervention.

## CRediT authorship contribution statement

**Weronika Szucio:** Writing – original draft, Methodology, Funding acquisition, Formal analysis, Data curation, Conceptualization. **Gabriela Milewska:** Methodology, Investigation, Formal analysis, Data curation, Conceptualization. **Michał Szamatowicz:** Methodology, Investigation, Formal analysis. **Weronika Lebiedzińska:** Methodology, Investigation, Formal analysis. **Oana Lupu:** Methodology, Investigation. **Piotr Bernaczyk:** Formal analysis. **Monika Leśniewska:** Investigation, Data curation. **Peilan Guo:** Investigation, Formal analysis. **Ilpo Huhtaniemi:** Writing – review & editing, Writing – original draft. **Xiangdong Li:** Validation, Supervision. **Sławomir Wołczyński:** Writing – review & editing, Writing – original draft, Supervision, Project administration. **Donata Ponikwicka–Tyszko:** Writing – review & editing, Writing – original draft, Supervision, Project administration, Methodology, Formal analysis, Data curation, Conceptualization. **Nafis A. Rahman:** Writing – review & editing, Writing – original draft, Supervision, Project administration, Conceptualization.

## Ethics declaration

The study was approved by the Local Human Investigation Ethics Committee (APK.002.274.2023). All procedures followed principles outlined in the World Medical Association's Declaration of Helsinki.

## Funding

This work was supported by a grant from the Medical University of Bialystok, Poland (No. B.SUB.24.231).

## Conflict of interests

None.
