# Supplementary material for: Progesterone and selective progesterone receptor modulator regulate tumor-associated fibroblasts in uterine leiomyomas
Source: Genes Dis. 2026 Jan 16;13(6):102041. doi: 10.1016/j.gendis.2026.102041 (PMC13380701; doi:10.1016/j.gendis.2026.102041)
Supplement: Multimedia component 1 [file mmc1.docx]

**Material & Methods**

**Study design**

The controlled laboratory study aimed to investigate the molecular mechanisms by which P4 and UA influence of the expression of various genes and proteins found in TAFs. To conduct transcriptional and functional analyses, we utilized tissue samples from patients with UL, and also UL explant in cultures. Fresh UL samples (n = 100) and healthy myometrial tissues (M, n = 30) were obtained immediately following surgical procedures conducted at the Department of Reproduction and Gynecological Endocrinology, Medical University of Bialystok, Poland. The Local Human Ethics Committee approved the study, and all participants provided written informed consent before surgery. The UL tissues were collected from women who underwent myomectomy due to infertility linked to ULs or who experienced severe clinical symptoms. Before surgery, patients were categorized into two groups: those who received UA treatment (UA-L group, n = 50) and those who did not (NT-L group, n = 50). For histopathological examination, all tissue samples were fixed in 10% buffered formalin for 48 hours, thoroughly rinsed with PBS (Gibco), dehydrated using a graded series of ethanol and xylene, and then embedded in paraffin blocks, using tissue cassettes. Fresh specimens were collected immediately post-surgery, preserved in RNAlater solution (Life Technologies) to prevent RNA degradation, rapidly frozen in liquid nitrogen, and stored at −80°C until further analysis.

**UL explant culture**

Immediately following surgery, ULs NT-L group were rinsed twice with PBS and sectioned into 1 mm fragments using a sterile scalpel on a Petri dish. Tissue explants were then placed in 24-well culture plates and incubated in basal DMEM/F12 medium (Gibco) supplemented with 10% fetal bovine serum (FBS; Gibco), 1% Antibiotic/Antimycotic Solution (Sigma–Aldrich), and 20 µL of plasmocin (Invivogen). Cultures were maintained at 37 °C in a humidified incubator with 5% CO₂. After 24 hours, explants were starved for 16 hours in phenol red-free DMEM/F12 containing 0.5% charcoal-stripped FBS and antibiotics. They were then exposed for another 24 hours to the following treatments in stimulation medium (phenol red-free DMEM/F12 with 0.5% charcoal-stripped FBS and antibiotic solution): ethanol vehicle (0.01%), P4 (1 µM), UA (1 µM), SMAD3 inhibitor (iSMAD3, 2 µM), transforming growth factor β3 (TGFβ3) (10 µM). UA, P4, and iSMAD3 (SIS3) were obtained from Sigma–Aldrich (St. Louis, MO, USA). TGFβ3 was obtained from R&D Systems. Following treatment, the culture media were collected for cytokine analysis, and explants were harvested for RNA isolation and gene expression profiling. Each condition was tested in eight independent experiments, performed in triplicate.

**Real-time quantitative qPCR**

Total RNA was initially extracted from UL following in vitro stimulation, as well as directly from UL tissues obtained from UA-treated and untreated patients, and from normal myometrial samples. RNA isolation was carried out using the TRIzol reagent (Invitrogen), following standard extraction procedures. The concentration and purity of RNA were assessed using a NanoDrop spectrophotometer (Thermo Scientific) and verified through gel electrophoresis. Reverse transcription (RT) was performed using the cDNA Reverse Transcription Kit with RNase Inhibitor (Applied Biosystems), in accordance with the manufacturer’s instructions. Quantitative real-time PCR (qPCR) was conducted using SYBR Green PCR Master Mix (Applied Biosystems) on a 7500 Real-Time PCR System (Applied Biosystems). The cycling conditions were: 2 minutes at 50 °C, followed by 10 minutes at 95 °C, then 40 cycles of 15 seconds at 95 °C and 1 minute at 60 °C. To confirm specificity, melting curve analysis and agarose gel electrophoresis were used to verify the amplification of single PCR products. All amplicons were further validated by sequencing. Gene expression data were normalized to the housekeeping gene Peptidylprolyl isomerase A (*PPIA*). The sequences of the primers were as follows: *αSMA* gene, F: GCCAAGCACTGTCAGGAATC, R: TTGTCACACACCAAGGCAGT; *PDGFRα* gene*,* F: GCCGCTTCCTGATATTGAGT, R: TGGATCTCCGTGATGATGTT; *PDGFRβ* gene*,* F*:* GAGGTGGTCAACTTCGAGTG, R: GATGGAGCGGATGTGGTAAG; *S100A4* gene*,* F: GGGCAAAGAGGGTGACAAGT, R: GTCCTTTTCCCCAAGAAGCTG; *syndecan-1* gene*,* F: GATGGAGGTCCTTCTGCCAC, R: TGAAGTCCTGCTCCCCAGAG; *FAP* gene*,* F: GCTGGGAATATTACGCGTCT, R: GCTCTTGCCATCACAGTTGA; *PPIA gene,* F: GCCAAGACTGAGTGGTTGGATG, R: GAGTTGTCCACAGTCAGCAATGG.

**Platelet-derived growth factor subunit B measurement**

Cytokine concentrations in explant culture supernatants were measured using the Human platelet-derived growth factor subunit B (PDGF-BB) Quantikine ELISA kit (DBB00; R&D Systems), according to the manufacturer’s protocol.

**Immunohistochemistry analysis**

UL tissues were fixed in paraformaldehyde and subsequently embedded in paraffin. For immunohistochemical analysis, paraffin sections were deparaffinized, rehydrated, and subjected to heat-induced antigen retrieval by boiling in 10 mM citrate buffer (pH 6.0) using a pressure retriever for 2.5 hours. To minimize non-specific binding, tissue sections were incubated with 3% BSA in PBS for 1 hour at room temperature. The primary antibody (Anti-syndecan-1 antibody, ab128936, Abcam, diluted 1:200) was incubated overnight at 4 °C. The following day, endogenous peroxidase activity was quenched by treating sections with 0.5% H₂O₂ in PBS for 20 minutes in the dark. Detection was carried out using Envision® anti-rabbit polymer conjugated to HRP (Dako), applied for 30 minutes at room temperature. Signal visualization was achieved using 3,3′-diaminobenzidine tetrahydrochloride (DAB; Dako). Between each step, sections were washed three times with PBS containing 0.05% Tween-20 (PBS-T). After DAB staining, sections were counterstained with hematoxylin, dehydrated, and mounted using Pertex mounting medium (Histolab Products AB, Askim, Sweden). Staining intensity in positively labeled cells was quantified as optical density (OD) using Fiji image analysis software. Five random fields per section were selected, and mean OD values were calculated for each area.

**Statistical analyses**

Data are presented as mean ± SEM. Statistical analysis was performed using one-way ANOVA followed by Bonferroni’s post hoc test or t-test, using GraphPad Prism version 7.0 (GraphPad Software, Inc.). A *p*-value of less than 0.05 was considered statistically significant.

**
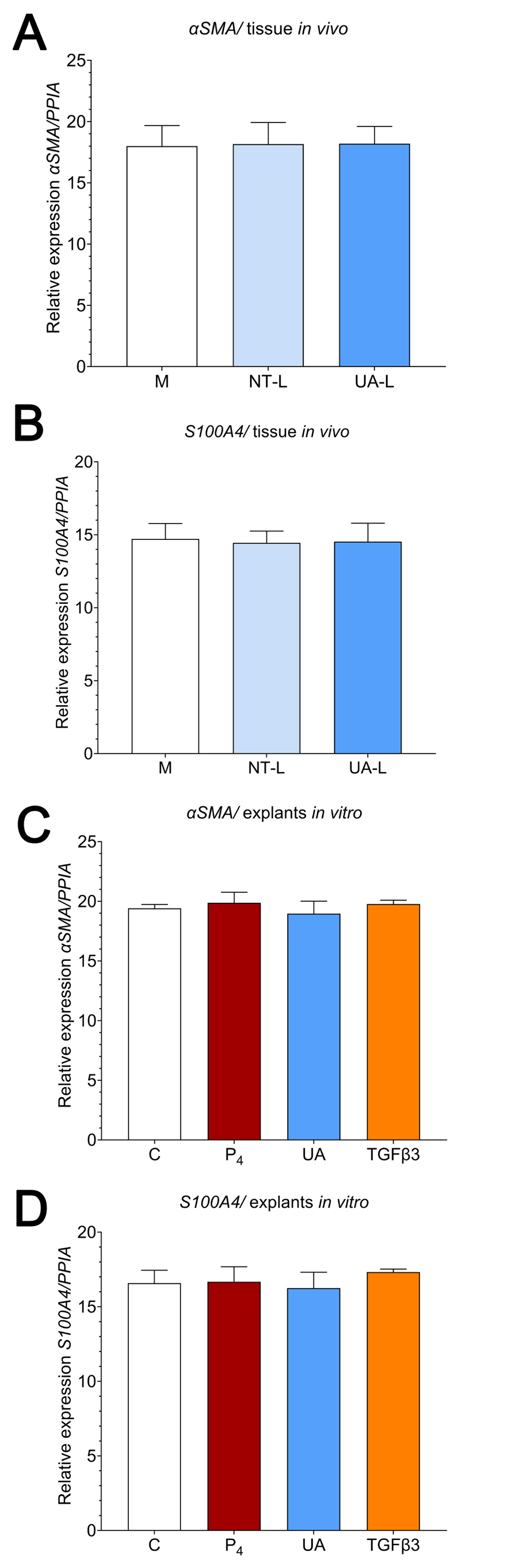
Fig S1**

**Figure legend**

**Fig. S1 TAF markers gene expression in UL tissue and explant culture**

The quantitative polymerase chain reaction (qPCR) gene expression analyses of alpha-smooth muscle actin (*αSMA*) and *S100A4* in healthy myometrium (M) (n= 30), uterine leiomyoma (UL) tissue after ulipristal acetate (UA) treatment (UA-L) (n=50) and non-treated group (NT-L) (n=50), Figs **A-B**; and UL explant culture *in vitro* treated with UA, progesterone (P4) or transforming growth factor beta 3 (TGFβ3), Figs **C-D**.

Statistical differences between groups were assessed using one-way ANOVA followed by Bonferroni’s post-hoc test. Bars labeled with different letters differ significantly (p < 0.05).

C = control; NT-L = nontreated ULs; M = normal myometrium; P4 = progesterone treatment; UA-L = ulipristal acetate treated ULs; TGFβ3 = transforming growth factor beta 3 treatment; UA = ulipristal acetate treatment; ULs = uterine leiomyomas
